# Investigating the Effect of Intrinsic Motivation on Alpha Desynchronization Using Sample Entropy

**DOI:** 10.3390/e21030237

**Published:** 2019-03-02

**Authors:** Tustanah Phukhachee, Suthathip Maneewongvatana, Thanate Angsuwatanakul, Keiji Iramina, Boonserm Kaewkamnerdpong

**Affiliations:** 1 Computer Engineering Department, Faculty of Engineering, King Mongkut’s University of Technology Thonburi, Bangkok 10140, Thailand; 2 Graduate School of Systems Life Sciences, Kyushu University, Fukuoka 819-0395, Japan; 3 Biological Engineering Program, Faculty of Engineering, King Mongkut’s University of Technology Thonburi, Bangkok 10140, Thailand

**Keywords:** sample entropy, ERSP, intrinsic motivation, attention, electroencephalography (EEG)

## Abstract

The effect of motivation and attention could play an important role in providing personalized learning services and improving learners toward smart education. These effects on brain activity could be quantified by EEG and open the path to analyze the efficiency of services during the learning process. Many studies reported the appearance of EEG alpha desynchronization during the attention period, resulting in better cognitive performance. Motivation was also found to be reflected in EEG. This study investigated the effect of intrinsic motivation on the alpha desynchronization pattern in terms of the complexity of time series data. The sample entropy method was used to quantify the complexity of event-related spectral perturbation (ERSP) of EEG data. We found that when participants can remember the stimulus, ERSP was significantly less complex than when they cannot. However, the effect of intrinsic motivation cannot be defined by using sample entropy directly. ERSP’s main effect showed that motivation affects the complexity of ERSP data; longer continuous alpha desynchronization patterns were found when participants were motivated. Therefore, we introduced an algorithm to identify the longest continuous alpha desynchronization pattern. The method allowed us to understand that intrinsic motivation has an effect on recognition at the frontal and left parietal area directly.

## 1. Introduction

Over the past decades, many countries have shown interest in “smart education” [1]. The core concept of smart education is to create an intelligent learning environment through smart technologies in order to suit learners at the individual level. In smart education, teaching methods using interactive online applications can provide personalized learning services and improve the individual learner. Most current services evaluate the efficiency of a method with the end result such as the improvement of learners through a testing system. However, if we could evaluate the effect of service to learners within their learning process, it could help us in adjusting the service to suit learners more efficiently so that smart education could help improve the learners at the individual level and also reduce the times that learners need to repeat the same services in the future.

Studies in cognitive neuroscience, which analyzed brain phenomenon resulting from stimulus directly to understand the underlying process of cognition, could allow us to analyze this learning process. In this field, electroencephalography (EEG) is one of the common methods to quantify the brain activity affected by stimulus directly. This method also has the advantage of flexibility so that it could be easily applied for use in different real-world environments. With this advantage, EEG becomes a powerful tool to provide brain activity information of individual learners during learning in their suitable environment. In the literature, it was found that the attention and motivation of learners were the crucial learning factors that affect the learning performance [2,3].

The relationship between attention measured by EEG and cognitive performance has been explored and analyzed in many research studies in cognitive neuroscience [4,5,6]. The results of these studies with the cognitive attention task reported better cognitive performance when the alpha frequency band of the EEG signal has negative values of the power spectrum compared with their baseline activity preceding the attention period [7,8]. This phenomenon is known as alpha desynchronization. To date, there have been various studies in both animals and humans related to attention tasks and alpha desynchronization.

Haegens et al. [9] recorded local field potentials and spikes from somatosensory, premotor, and motor regions of a trained monkey with a vibrotactile discrimination task to investigate the reason behind decreased oscillatory alpha activity related to neuronal firing rates. They found a decrease in power of the alpha band in the sensorimotor regions during the discrimination task, which could be used in predicting better discrimination performance. They also found that neuronal firing rates increase with decreased alpha power. With these findings, they suggested that alpha oscillations exercise a strong inhibitory influence on spike timing and firing rate. These findings could clarify the hypothesis from some previous studies [10,11] at the neuronal level that the increased alpha activity is related to the attention task and functions in suppressing other distracting inputs in task-irrelevant regions.

The desynchronization in the alpha band is also confirmed to be found in the attention period in healthy human subjects, but missing in traumatic brain injury (TBI) subjects who have cognitive problems. The work of Babiloni et al. [4] is an example study on healthy human subjects with a visual cognitive experiment. Babiloni et al. [4] reported that the decreases in power from the alpha band of participants were found in the attention period of the experiment. However, Dockree et al. [5] found no evidence of this alpha desynchronization phenomenon in traumatic brain injury subjects with a fixed sequence sustained attention to response task. They suggested that these patients may have dysfunctional alpha generators due to their injuries, which lead to increased lapses of attention and affect their performance during the task. In addition, speech perception could also have similar brain cognitive function as attention visual stimuli on human subjects. Krause et al. [6] conducted an auditory memory scanning paradigm experiment and found the pattern of desynchronization in the alpha brainwave band of their participants. From their results, they suggested that event-related desynchronization (ERD) in the alpha band is closely associated with higher cortical processes such as memory functions.

Intrinsic motivation is another aspect that could improve the cognitive performance of the individual learners. This intrinsic motivation is the self-determination of the subject with respect to the task such as the feeling that the task is interesting or feeling proud when the subject can complete the task, so the subject pays more attention to completing the task. This phenomenon was also found to affect the EEG signal. In the work of Jia et al. [12], participants did the “interesting stop-watch task”/“boring-watch stop task”. From their result, they suggested that these interesting and boring intrinsic motivations of humans can be detected at the neuron level, which could be quantitatively indexed from ERP components (N2, the feedback-related negativity (FRN), P300). Banerjee et al. [13] also found that participants had better performance when targets were more interesting in the visuospatial task with interesting/uninteresting targets. The intrinsic motivation was found to influence endogenous attention (anticipation) [13], which could be observed in EEG electrodes within the parieto-occipital area.

From the literature, we know that intrinsic motivation could affect attention [13], that attention could be observed with alpha desynchronization in the EEG signal [4,6] and that alpha desynchronization could be used to predict the cognitive performance of participants in cognitive attention task [7,8]. However, an in-depth study on the effect of intrinsic motivation behavior on the alpha desynchronization phenomenon is still missing. The extensive study on this intrinsic motivation, attention, and alpha desynchronization relationship could provide additional valuable information to the field. For example, the finding of the unmotivated characteristic in cognitive data of the learners could later be used as another important factor in consideration when evaluating suitable teaching and learning methods, as well as a better environment to improve the learning efficiency of participants. In this study, we aimed to investigate the effect of intrinsic motivation on alpha desynchronization for each cognitive result of participants and identify the area of electrodes that get affected.

In our previous study [14], we explored the differences between the attention period and the decision period in a visual cognitive experiment. We found that 85% (averaged from five participants) of ERSP data during the attention period in the alpha frequency band have a low power spectrum, whereas only 64% of ERSP data have a low power spectrum in the alpha band during the decision period. Such a frequent occurrence of the low power spectrum in a duration of time could lead us to investigate further in terms of complexity in time series of brain activity data. In this study, we used sample entropy to quantify the complexity of brain activity in order to identify the effect of motivation on alpha desynchronization. Sample entropy has been introduced specifically for the analysis of non-stationary physiological signals by capturing the amount of self-similarity within time series of interest [15]. The method has an advantage in handling the noisy time-series data, which is the characteristic of physiological data [15]. In addition, sample entropy is suitable for the shorter time-series dataset, which is appropriate for the task with human subjects [16]. The main hypothesis in this study is that when participants are paying attention to the task, the data during the attention period will be less complex due to the frequent appearance of alpha desynchronization pattern.

## 2. Materials and Methods

**Figure 1:**
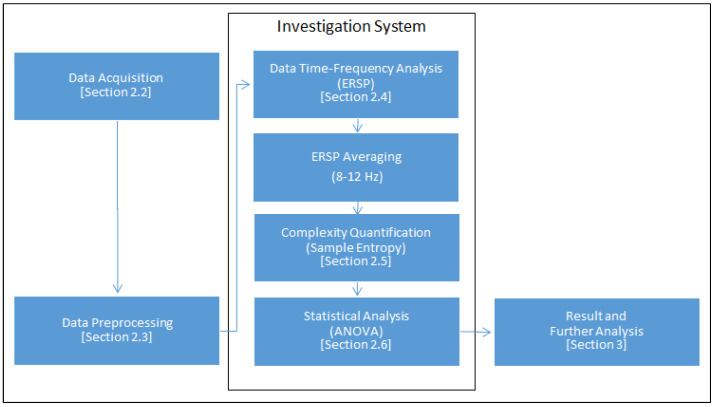
The process used for investigating the effect of motivation on EEG data in this study.

To test our hypothesis, we conducted a cognitive experiment with a visual memory task followed by a recognition test. EEG data were collected during the experiment. Participants could voluntary decide whether they were motivated to remember the stimulus or not. The recognition test allowed us to investigate how the intrinsic motivation of participants could be related to their cognitive performance. The power spectrum complexity of these data was then analyzed to attest our hypothesis. EEG should yield different power spectrum complexity for each cognitive result. Sample entropy was used to quantify the complexity of time-frequency domain power spectrum data. This time-frequency domain power spectrum data were transformed from EEG data by the event-related spectral perturbation (ERSP) method. The process used for investigating the effect of motivation in this study is shown in Figure 1.

### 2.1. Participants

We performed the cognitive experiment with sixteen volunteer participants with consent. The participants were adult with mixed gender, aged between 21 and 37. None of the subjects had prior visual perception or memory disorders.

### 2.2. Stimuli and Procedure

**Figure 2:**
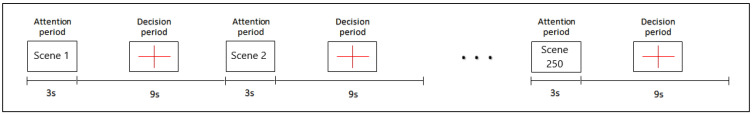
The experimental procedure in the cognitive experiment to gather EEG data in this study.

To study the alpha desynchronization characteristic of the motivation effect on cognitive performance, we divided the experiment into two parts: cognitive experiment and recognition test. In the cognitive experiment, participants were presented with random unique visual stimuli, which were indoor/outdoor scenes from the Scene UNderstanding (SUN)database [17]. Each stimulus remained on the screen for 3 s after being presented, then changing to the fixation screen. This period will later be referred to as an attention period. The fixation screen was shown for another 9 s. During this period, the participant was allowed to make his/her decision whether they would like to remember the scene or not freely. This period will be referred to as a decision period. The response was made by clicking mouse buttons: the left one for the case where they wanted to remember the scene and the right one for the case where they did not want to remember it. There was no constraint in their decision; hence, the number of responses in this cognitive experiment could be unequal. After the decision period, the screen then changed to another scene. This procedure is presented in Figure 2.

**Figure 3:**
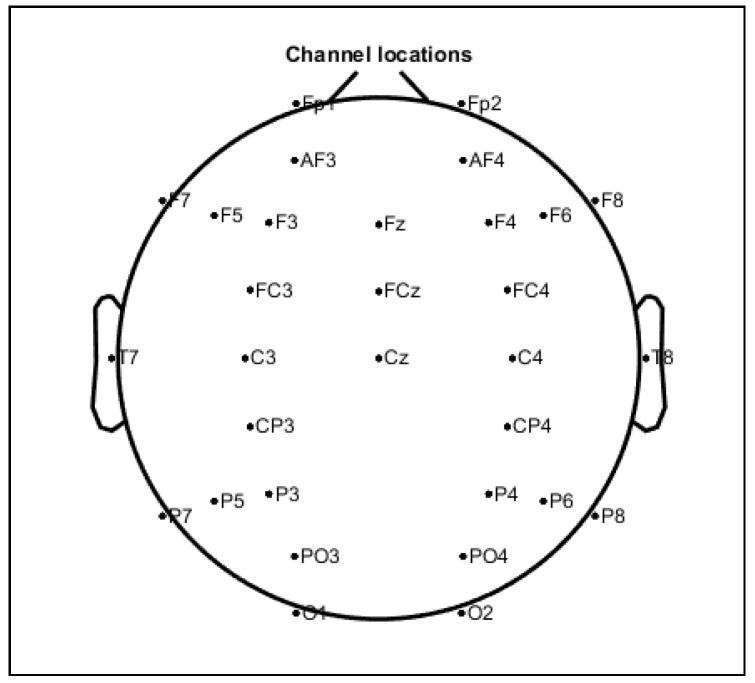
The EEG montage of the recordings used in this study.

After 250 scenes were presented, the cognitive experiment ended. The participants rested for 10 min. The scene recognition test was then started. In the recognition test, 500 random scenes comprised of 250 scenes from the cognitive experiment and 250 new scenes were presented one by one. The participants answered whether they could recognize the scenes in the cognitive experiment or not. This experiment was similar to the study of fMRl by Yoo et al. [18]. During the cognitive experiment, EEG data were collected at a 500-Hz sampling rate from 31 EEG electrodes recording at the location illustrated in Figure 3. The access to the dataset used in this study can be found in Supplementary Materials. The EEG equipment used to obtain data in this study was Nihon Kohden Neurofax EEG-1100 (Nihon Kohden, Tokyo, Japan).

### 2.3. Preprocessing

After collecting raw data from the cognitive experiment, the EEG signals were analyzed by using MATLAB (R2014b) with open source toolbox EEGLAB v13.4.4b [19]. We first mapped the data to their corresponding electrode locations. Average referencing was used in this study. The “average referencing” was conducted by using the reref function from the EEGLAB toolbox. The method subtracts the average potential of all electrodes from each electrode for each time point. The bandpass filter of 0.5–50 Hz was used to remove noises. A notch filter of 60 Hz was then applied to remove physiological and power line noise, respectively. After that, the signals were marked into epochs corresponding to their responsive event regarding stimulus onset. Each epoch contained data from 1 s before the stimulus presented to 12 s after the stimulus appeared. The epochs that had a voltage higher than 500 microvolts or lower than −500 microvolts were considered as abnormal value epochs and disregarded in this study. The epochs were then analyzed for independent components to remove components related to eye blinking artifacts. Lastly, the epochs that had a saccade characteristic were also discarded. In addition, the epochs with no response received within the decision period were excluded from this study. All remaining epochs were then computed for ERSP.

The data epochs of each subject could be categorized by their motivation into two groups: want to remember and do not want to remember cases. As stated in the previous section, since the subjects could make their decision freely upon their motivation, the number of epochs in each category could be different. Furthermore, the scene recognition test could give us more information on each category and allow us to identify whether the participants recognized the scenes regarding their response in the cognitive experiment or not. By considering the cognitive result of their motivation in the recognition test, the epochs were further categorized into four groups: want to remember and remembered, want to remember, but forgot, do not want to remember, but remembered, and lastly, do not want to remember and forgot. In this study, we refer to them as RR, RF, FR, and FF, respectively. The first part of each group represented their motivated decisions in the cognitive experiment while the latter part indicated their response in the recognition test. For example, in the RR case, this group represented the case where participants decided to remember the scene in the cognitive experiment and could later recognize it in the recognition test.

### 2.4. Event-Related Spectral Perturbation

After the preprocessing steps of the data, we then quantified the power spectrum of the preprocessed data relative to their corresponding baseline activity preceding the stimulus appearing in order to identify the alpha desynchronization pattern occurring in each case of our task. In this study, the data from −1000 ms–0 ms of the stimulus onset served as the baseline. The method we used in this process was baseline-normalized event-related spectral perturbation (ERSP). ERSP is a method of measuring the event-related average dynamic changes in the amplitude of the EEG frequency spectrum. The method was introduced by Makeig [20], which later was implemented in the EEGLAB toolbox [19]. The ERSP method starts with computing the power spectrum over sliding latency windows of each epoch and then averaging across the epoch afterward. Hence, each data point of the result indicates the EEG signal spectral power of a given frequency and latency relative to a sliding window in dB. The transformation equation is shown in (1).

(1) $$ERSP\left(f,t\right)=\frac{1}{n}\sum_{k=1}^{n}{\left|F_{k}\left(f,t\right)\right|}^{2}$$

The equation represents the calculation of the ERSP value, which is the average spectra of *n* epochs at each frequency and time window. *n* usually is the number of identical category epochs. Each epoch *k* in *n* epochs is computed for the spectrum of frequency *f* of the time window centered at time point *t* by using function $F_{k}\left(f,t\right)$. For the default option, EEGLAB performs frequency transformation of $F_{k}\left(f,t\right)$ by using fast Fourier transform (FFT). After the calculation of ERSP, the baseline-normalized ERSP is then computed. This process was done by subtracting the mean baseline power spectrum from the ERSP at each frequency and time window.

After the ERSP step, we now have the data from 31 channels of 16 participants and four types, each with the time series data of 101 time points. We then averaged the ERSP result across the alpha frequency band (8–12 Hz) at each channel based on the time point; at each time point, ERSP values of frequencies of 8 Hz–12 Hz were averaged. This step is essential because alpha desynchronization of each individual could occur in a different frequency.

### 2.5. Sample Entropy

After we obtained the averaged data of alpha frequency ERSP with 101 data points for each subject, each channel, and each type of interest, we quantified the complexity of these data with the sample entropy method. Sample entropy is a measure of the complexity of time-series data. The higher sample entropy value indicates more irregularity, as well as its complexity in the data and vice versa. Specifically, sample entropy is the negative natural logarithm of the conditional probability that two sequences of *m* points similarly within a tolerance value remain similar at the next point, m+1, within the same tolerance value when excluding self-matches in calculating the probability. The method of sample entropy is the modification of approximate entropy [21] introduced by Richman and Moorman [15] to reduce the bias from self-matching that could occur in approximate entropy. In general, sample entropy is calculated by Equation (2):

(2) SampEn(m,r,N)=-ln(A/B)

where *N* denotes the number of data points in time-series data, *m* denotes the template pattern size, *r* denotes the tolerance rate to consider the similarity of the data, and *A* and *B* denote the number of pattern matches for the pattern of lengths m+1 and *m*, respectively. The tolerance rate is often set to a certain percentage of the time-series standard deviation (SD) [15]. We selected 0.2 multiplied by SD as the tolerance rate as recommended by [15]; [15] reported that for the shorter data, an N=100, 0.2 SD tolerance rate is the smallest value, with the most precise similarity, which can help sample entropy to measure the similarity of uniform distribution data and give the sample entropy value according to the theoretically-predicted value.

### 2.6. Statistical Analysis

We used the analysis of variance (ANOVA) to test the hypothesis that there are differences in sample entropy on the ERSP data with different factors of interest. The factorial design of the experiment with a 5% significance level was conducted with the number of replications equal to the number of participants. There were three factors including motivation types, recognition types, and electrode channels. The motivation types were separated into “want to”/“do not want to” remember categories. The recognition types were separated into the remembered and forgot categories. The channel factor was separated into 31 electrode channels. The sample entropy value of ERSP averaged across the alpha frequency band during the attention period is the response.

## 3. Results and Discussion

After preprocessing, we had 3044 epochs of preprocessed EEG data for each channel from 16 participants. After separating into four categories based on the motivation of participants and their recognition results, there were 1094 epochs in the RR case (participants want to remember and could remember the scene), 332 epochs in the RF case (participants want to remember, but later forgot the scene), 1092 epochs in the FR case (participants do not want to remember, but could later remember the scene), and 526 epochs in the FF case (participants do not want to remember and forgot the scene).

### 3.1. ERSP Main Effect of Motivation and Recognition

**Figure 4:**
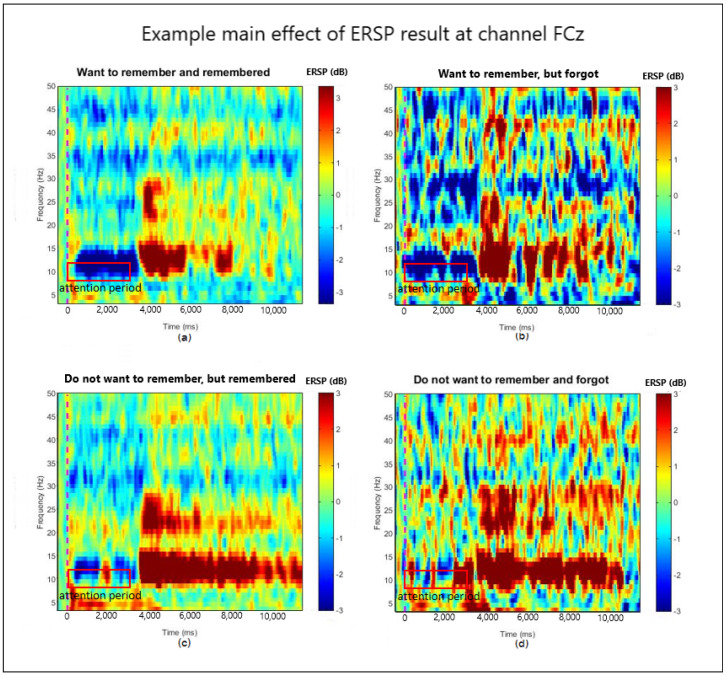
Example of motivation and recognition main effects at channel FCz (the *x*-axis is a time value in milliseconds, and the *y*-axis is a frequency value in Hz): (**a**), (**b**), (**c**), and (**d**) are from the RR (R, remember), RF (F, forget), FR, and FF cases respectively. The attention period starts when the stimulus is presented (zero milliseconds) and ends at 3000 milliseconds, where the stimulus disappeared.

The preprocessed EEG data in each category were then transformed into the dynamic EEG frequency spectrum with ERSP. For each channel of each participant, the main effects of motivation and recognition in all categories were analyzed based on ERSP values for the main characteristic of each case. An example of the ERSP main effects from channel FCz of a participant plotted in the time-frequency scale is presented in Figure 4: (a), (b), (c), and (d) are from the RR, RF, FR, and FF cases, respectively.

From this result, we now have some insights on our dataset. We noticed that, among motivation-recognition cases, there were differences in the characteristic of desynchronization in the alpha band, 8–12 Hz [22], in the attention period during stimulus presentation in the first 3 s. The phenomenon where the presence of continuous alpha desynchronization pattern was found in all cases could be the result of where the participants had to pay attention to the stimulus to make their decision whether they were motivated or not. However, in the case where participants wanted to remember the stimulus (a) and (b), they would likely pay more attention than when they did not want to remember the stimulus. This attention should be the cause of longer continuous patterns of alpha desynchronization appearing in the attention period (0–3 s); therefore, the power spectrum in the attention period should be less complex than the usual on-going signal of the participants. In the case where participants wanted to remember the scene and could later remember the scene in their recognition test (RR), the presence of continuous alpha desynchronization within the attention period was usually wider than other cases. There was a small variation in the value of ERSP in this period. On the contrary, in the case of participants not wanting to remember the scene or they could not remember the scene in the recognition test, the ERSP data were more complex than the RR cases. This finding leads us to focus the analysis of this study on the complexity of the alpha band in the attention period.

### 3.2. Sample Entropy Analysis of ERSP

We hypothesized that the complexity of ERSP data during the attention period would be different among the four cases in our study. The complexity of ERSP data was measured by the sample entropy method. We then used ANOVA to analyze the factors that could influence the data of participants, which resulted in different complexity among the four cases. There were three factors tested here: the motivation of the participant (“want to”/“do not want to” remember), the recognition result of the participants after the experiment (remembered, forgot), and channels (31 electrodes). A sample entropy value of a pattern size equal to two was used as a response in this test. Our null hypotheses was that the different condition of each main factor caused no difference in the sample entropy values. We also verified the normal probability assumption of our ERSP sample entropy data. The result indicated that our data were normally distributed.

**Figure 5:**
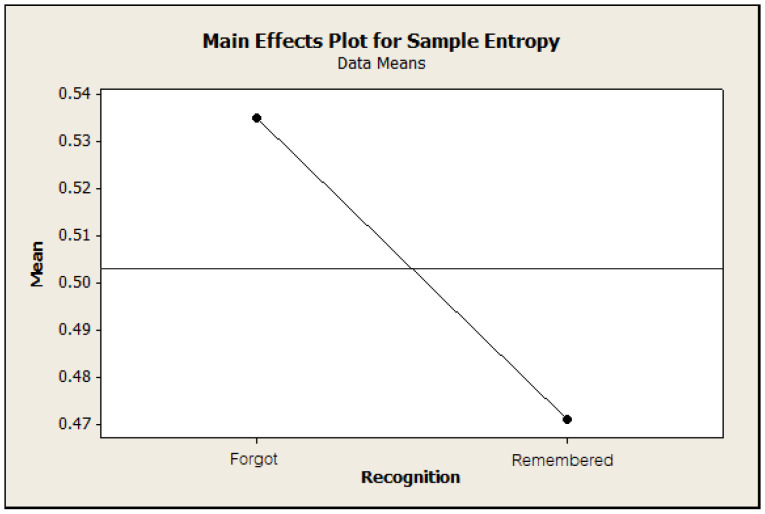
The main effects plot of the recognition test factor with sample entropy values of the pattern size equal to two as responses.

The *p*-value of each declared factor was 0.006 for channels, 0.305 for motivation, whereas the *p*-values for the recognition result were lower than 0.001. These results indicated that the recognition result and channels were rejected by the null hypothesis with a *p*-value lower than 0.05. Therefore, there was strong evidence to conclude that the different channels and recognition results factors had different effects on the sample entropy values. We presented the main effect of the recognition result on sample entropy, which indicated that the forgot cases had higher sample entropy values than the remembered cases in Figure 5.

The same result was also confirmed by performing a paired *t*-test. We tested this by using paired *t* analysis of all sample entropy values between these two cases of recognition results in the same participant with the same channel and motivation type. We assigned the null hypothesis as there was no difference between the mean sample entropy of both cases of recognition results and alternative hypothesis as there was lower mean entropy values for the case that the participants could remember the stimulus than for the case that they could not remember the stimulus. The result of this test stated a *p*-value lower than 0.001. With this result, we have a strong conclusion that the remembered cases of each participant had significant lower sample entropy values than the forgot cases.

**Figure 6:**
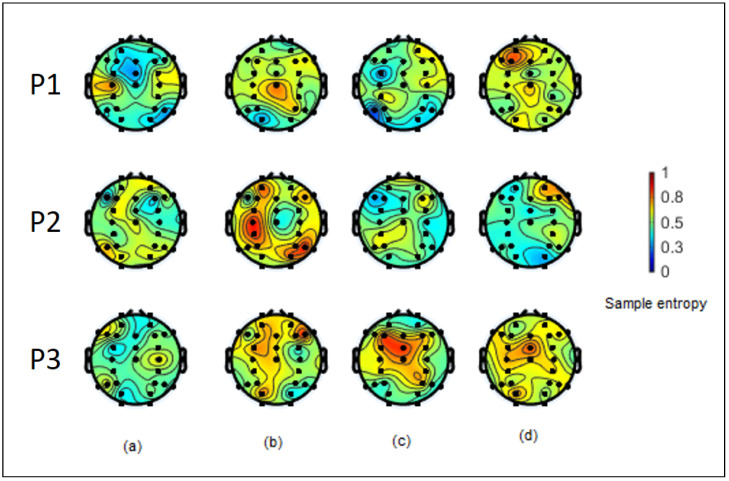
Example of sample entropy values with a template size equal to two mapped onto head models of three participants: (**a**), (**b**), (**c**), **d**) are from the RR, RF, FR, and FF cases, respectively.

With all the above results, the complexity measurement of sample entropy can help us differentiate the cognitive performance of the participants in our experimental results. Even though the ANOVA result indicated that there was at least one channel that had a significant difference in sample entropy compared to other channels, with 31 channels and the randomness of the sample entropy among them, we could not clearly differentiate which channels were the main channels that were affected by the stimulus. We present the examples of this randomness of sample entropy mapped onto the head model of three participants in Figure 6.

### 3.3. Continuous Alpha Desynchronization Pattern

The ERSP main effect of motivation and recognition allowed us to observe the differences in the continuous pattern of alpha desynchronization; when the participants were motivated, the longer alpha desynchronization pattern was observed in the attention period. The classical sample entropy with a template size equal to two allowed us to confirm that when the participants remembered, they had lower sample entropy values than when they forgot. However, the classical sample entropy with the template size equal to two could not help in differentiating the complexity between the motivated and unmotivated cases and could not help to pinpoint the channel location affected by the stimulus.

From Figure 4, the differences between motivated and unmotivated cases can be observed in the pattern of alpha desynchronization. We noticed that when participants were motivated, there appeared to be a long continuous pattern of alpha desynchronization; some non-uniform positive alpha values rarely appeared in the case where participants could remember the scene (a), whereas there appeared to be more non-uniform positive alpha values of the ERSP data when participants could not remember the scene (b). This finding resulted in different complexity in ERSP alpha pattern between two recognition cases. On the contrary, when participants did not want to remember the scene, the ERSP data appeared to be complex in both the remembered and forgot cases (c, d); however, there appeared to be more continuous pattern of alpha desynchronization when participants could remember the scene (c), but a more continuous pattern of positive alpha values appeared when participants could not recognize the scene afterward (d). Hence, both cases could result in having similar complexity after applying the sample entropy method (*m* = 2). This observation could help us differentiate between motivation cases by considering motivation and recognition parts concurrently.

**Figure 7:**
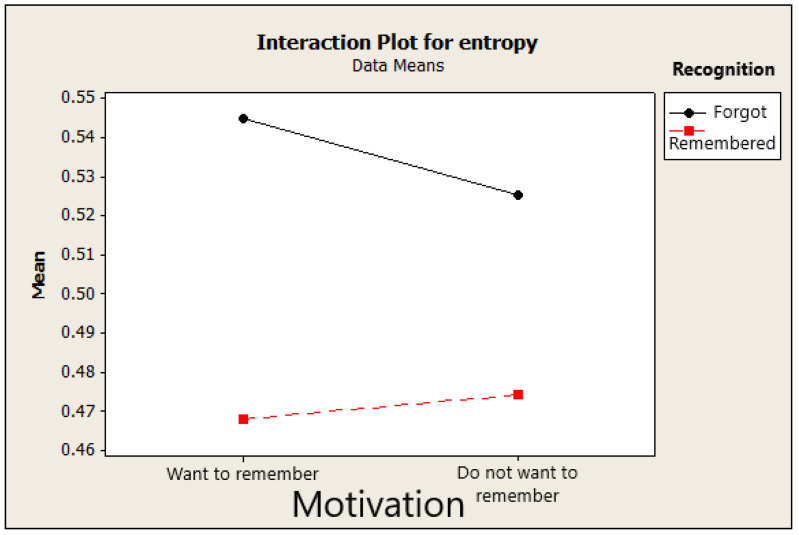
The interaction between motivation and recognition result factors.

This observation is supported by the interaction effect of the previous factorial experiment. We also found the interaction between motivation factor and recognition result factors with *p* = 0.048, as shown in Figure 7. The interaction between these two factors indicated that the effect of the recognition result on the sample entropy value depends on the levels of motivation factor. The average sample entropy for the forget case was much higher than the remembered case when the participants wanted to remember the stimulus, whereas there was a smaller difference between the values of forgot and remembered cases when the participants did not want to remember the stimulus.

It could be hypothesized that when the participants are unmotivated, the complexity of ERSP from both recognition cases would have much more similarity than in the case where the participants are motivated. This hypothesis could be tested by comparing the complexity between remembered and forget cases’ data within each motivation case separately. This complexity was determined by using sample entropy, which was calculated from Equation (2), with a template size equal to zero; in this case, *A* is equal to the number of matches with a pattern length of one, while *B* is equal to the number of all possible patterns with a length of one. The *B* value can be calculated by n×(n-1)/2, where *n* is the length of input data. Note that this calculation is focused on the pure complexity of the data rather than on the complexity of the continuous temporal pattern in the previous section where the size of *m* was equal to two. The paired *t*-test was used to identify this significant difference of complexity between the remembered and forgot cases. The null hypothesis here was that the two cases were similar to one another. The null hypothesis will be rejected at the 5% significance level.

In order to identify the channels that are affected by the stimulus, we hypothesized that the main channels affected by the stimulus should have longer continuous patterns of alpha desynchronization value in the remembered case than the forgot case. The longer continuous pattern reflects the increase of its sub-pattern. For example, if data of a size of 5 have 4 similar values, 11110, then the data contain 1 template match of a length of 3 (111xx matched with x111x), 3 matched patterns of a length of 2 (11xxx matched with x11xx, 11xxx matched with xx11x, and x11xx matched with xx11x), and 6 matched patterns of a length of 1. However, if the data have 5 similar values, 11111, then they will contain 1 template match of a length of 4, 3 matched patterns of a length of 3, 6 matched patterns of a length of 2, as well as the 10 matched patterns of a length of 1.

We tested this hypothesis by introducing the use of testing the significant difference of sample entropy between two recognition cases with an incremental continuous template pattern size. We presented the algorithm to identify the longest size of continuous alpha desynchronization pattern that can distinguish the remembered case from the forgot case of each motivation case in Algorithm 1.
**Algorithm 1** Identifying the longest continuous alpha desynchronization pattern.1: template_size = 0 2: r_remem = 0.2*SD of input_data_remem 3: SE_remem = sampEn(input_data_remem, template_size, r_rate) 4: r_forgot = 0.2*SD of input_data_forgot 5: SE_forgot = sampEn(input_data_forgot, template_size, r_rate) 6: While *p*-value of paired *t*-test between SE_remem and SE_forgot > 0.001 do 7:    template_size = template_size+1 8:    SE_remem = sampEn(input_data_remem, template_size, r_rate) 9:    SE_forgot = sampEn(input_data_forgot, template_size, r_rate) 10: End while

**Figure 8:**
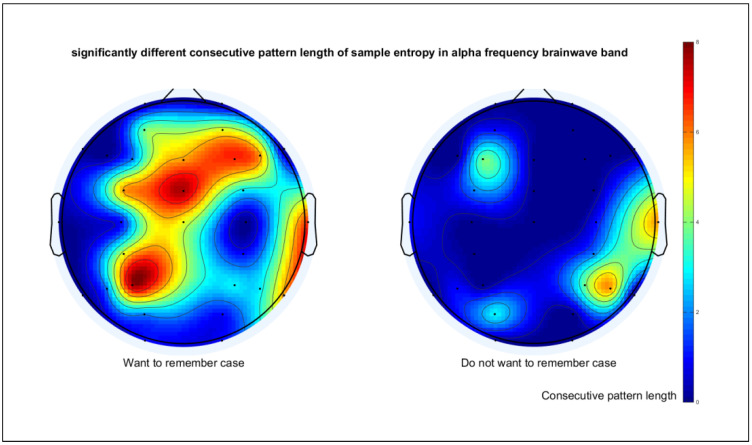
The plots of the longest alpha desynchronization pattern mapped to corresponding channel locations on the head model; the left head model is the case where participants want to remember the stimulus; the right model is the case where the participants do not want to remember.

In this study, we first determined the longest significantly different pattern by using this algorithm separately for each motivation case. The input data were the sample entropy of ERSP data from all 16 participants. For each motivation case, the sample entropy of remembered and forgot cases with an incremental continuous template pattern size was compared. The template pattern size started from zero and, then, increased for one size for the new comparison until the null hypothesis was rejected. The resulting longest consecutive template pattern size with the significant difference in sample entropy value was then mapped to the head model corresponding to the channel locations, as presented in Figure 8.

Figure 8 illustrates that with motivation, the complexity patterns of ERSP were different; the main channels affected by the stimulus included channels around the frontal area such as FCz and F4 and the left parietal area such as P3. The affected channels also coincided with the conventional channels related to the attention task. The frontal area is one of the conventional areas used in EEG cognitive research, which was found to be related to the attention and cognitive performance of subjects [23,24]. The cognitive task with scenery stimulus was also found to cause the activation of the parahippocampal place area (PPA) in the temporal area in fMRI research [25,26,27]. This study also provided important confirmation of EEG on the findings in the fMRI study of [18], which pointed out the significant difference in left PPA activation between the remembered case and forgot case. Their research also claimed that there was no reliable difference in right PPA activation.

The result in Figure 8 also suggests that when participants do not want to remember the stimulus, the ERSP values in the alpha band appeared to have similar complexity in both recognition cases. The similarity in randomness from unmotivated participants could cause ambiguous results and may result in a misleading interpretation and wrong conclusion for attention task-related studies. Therefore, we suggested excluding these data before assessing the stimulus, teaching, and learning methods in the future. Since our investigation method required a sufficient number of epochs to determine the motivation of participants and suitable electrode placement, it is still not suited for online application and could only be used as an offline method to have an overview of the data before further analysis.

The findings in our study showed the global characteristic differences of alpha desynchronization patterns under intrinsic motivation that are reflected through the complexity of the data in the attention period of the experiment. The findings suggested that the effect of intrinsic motivation could influence the participants’ cognitive result. This insight led us to believe that providing a sufficient dataset to the current popular feature learning method, deep learning [28], could potentially make the online classification of the individual learner’s motivation possible, which could lead to a better smart education system in the future.

## 4. Conclusions

By conducting a cognitive experiment with a visual memory task followed by a recognition test, this study investigated the effect of intrinsic motivation on alpha desynchronization for each recognition case of participants. Based on the complexity of ERSP data in the alpha band, we used sample entropy with a template size equal to two and found that in the case where participants can recognize the stimulus, the ERSP data are less complex than when participants cannot. The result from ANOVA indicated that at least one electrode has effects on sample entropy values. We then introduced the algorithm to identify the longest continuous alpha desynchronization pattern that can significantly distinguish the remembered case from the forgot case of each motivation choice. The result suggested that when participants are unmotivated, the complexity of the data between two recognition cases is similar. The areas where we can observe the difference the most are the frontal and around the left PPA areas, which are also consistent with the findings in previous research [18,23,24,25,26,27]. These findings allowed us to grasp the global characteristic differences of alpha desynchronization patterns under intrinsic motivation and the locations to place electrodes for detection. These findings could be useful in assessing the efficiency of stimulus, teaching, and learning methods so that we could better improve the quality of learning in smart education in the future.

## Supplementary Materials

The dataset used in this study is available online at http://www.bioeng.kmutt.ac.th/en/research/research-areas/computational-bioengineering/18-category-en/computational-bioengineering/167-investigating-the-effect-of-intrinsic-motivation-on-alpha-desynchronization-using-sample-entropy.
